# Complete Genome Sequence of a Variant of the Methicillin-Resistant *Staphylococcus aureus* ST239 Lineage, Strain BMB9393, Displaying Superior Ability To Accumulate *ica*-Independent Biofilm

**DOI:** 10.1128/genomeA.00576-13

**Published:** 2013-08-08

**Authors:** Maiana Oliveira Cerqueira Costa, Cristiana Ossaille Beltrame, Fabienne Antunes Ferreira, Ana Maria Nunes Botelho, Nicholas Costa Barroso Lima, Rangel Celso Souza, Luiz Gonzaga Paula de Almeida, Ana Tereza Ribeiro Vasconcelos, Marisa Fabiana Nicolás, Agnes Marie Sá Figueiredo

**Affiliations:** Laboratório Nacional de Computação Científica, Laboratório de Bioinformática, Petrópolis, Rio de Janeiro, Brazila; Universidade Federal do Rio de Janeiro, Instituto de Microbiologia Professor Paulo de Góes, Departamento de Microbiologia Médica, Rio de Janeiro, Brazilb

## Abstract

Biofilm is considered an important virulence factor in nosocomial infections. Herein, we report the complete genome sequence of a variant of methicillin-resistant *Staphylococcus aureus*, strain BMB9393, which is highly disseminated in Brazil. This strain belongs to the lineage ST239 and displays increased ability to accumulate *ica*-independent biofilm and to invade human epithelial cells.

## GENOME ANNOUNCEMENT

*Staphylococcus aureus* subsp. *aureus* strains are highly adaptive and versatile Gram-positive bacterial isolates (1, 2). Methicillin-resistant *S. aureus* (MRSA) bacteria displaying high-level multiresistance are of great concern worldwide (3). In Brazil, a multiresistant *S. aureus* clone (Brazilian epidemic clone [BEC]) of the lineage ST239-SCC*mec*III is widely disseminated in hospitals. ST239 isolates have also spread to other South American countries and countries in Europe, Asia, and Oceania (4–9). Here, we report the complete genome sequence of an ST239 variant, strain BMB9393, which displays superior ability to accumulate *ica*-independent biofilm and to adhere to and invade human airway cells. This variant was isolated in 1993 from a case of nosocomial bloodstream infection in Rio de Janeiro (4).

The genome sequencing was performed using a 454 GS FLX titanium (3-kb paired-end library) approach (Roche Diagnostics Corporation). The assembly, based on 285,317 reads corresponding to 102,585,816 bp (29-fold coverage), was carried out using Newbler v 2.6 (Roche) and Celera genome assembly v 6.1 (JCV Institute). Gaps within scaffolds resulting from repetitive sequences were resolved by *in silico* gap filling.

The genome of BMB9393 consists of one circular chromosome with 2,980,548 bp (GC content of 32.92%) and one circular plasmid of 2,908 bp (pBMB9393). Using the Sabia pipeline (10), we performed functional annotation of 2,678 protein-coding sequences (CDS), among which 2,244 were assigned to known functions and 434 were of unknown categories. The genome harbors 5 rRNA operons (5 copies of 16S rRNA, 5 of 23S rRNA, and 6 of 5S rRNA) and 60 tRNA genes, which were identified with RNAmmer and tRNAscan, respectively. Additionally, 75.46% of the CDS were assigned to at least one COG group.

The comparative analyses were performed using the MaGe MicroScope platform (11). The comparison (50% amino acid identity, 80% alignment coverage) with three other published genomes of ST239 *S. aureus* revealed that BMB9393 shares 2,579 CDS with strain JKD6008, 2,555 with strain TW20, and 2,541 with strain T0131. BMB9393 has 142 unique open reading frames (ORFs) compared with the other three genomes, from which 14 were associated with virulence and antimicrobial resistance. pBMB9393 is similar to pC194, carries the *cat* gene encoding chloramphenicol resistance, and is not found in the other three genomes. Approximately 14% of the BMB9393 chromosome corresponds to regions of genomic plasticity (RGPs). Three RGPs are phage related, including phage φNM3, which carries the virulence genes *sak*, *scn*, and *chp*, encoding staphylokinase, staphylococcal complement inhibitor, and chemotaxis-inhibitor protein, respectively. BMB9393 also harbors the genomic island *v*SAα, which carries lipoproteins and superantigen gene clusters, and *v*SAβ (absent in JKD6008), which harbors genes associated with antibiotic and toxin production, as well as a cluster of genes encoding serine proteases. Finally, BMB9393 carries a staphylococcal pathogenicity island (SAPI) that is a structural mosaic between SAPI1 and SAPI2. BMB9393 has a number of RGPs associated with insertion elements (IS) and transposons (Tn), including 15 copies of IS*256*, four of Tn*554*, and two of Tn*552*.

### Nucleotide sequence accession numbers.

The complete genome data of BMB9393 have been deposited in GenBank with the accession numbers CP005288 for the chromosome and CP005289 for the plasmid.
